# R(+)-Thioctic Acid Effects on Oxidative Stress and Peripheral Neuropathy in Type II Diabetic Patients: Preliminary Results by Electron Paramagnetic Resonance and Electroneurography

**DOI:** 10.1155/2018/1767265

**Published:** 2018-04-10

**Authors:** Simona Mrakic-Sposta, Alessandra Vezzoli, Luca Maderna, Francesca Gregorini, Michela Montorsi, Sarah Moretti, Fulvia Greco, Emanuela Cova, Maristella Gussoni

**Affiliations:** ^1^Institute of Bioimaging and Molecular Physiology, National Council of Research (CNR), Segrate, Italy; ^2^Istituto Auxologico Italiano, Milan, Italy; ^3^Telematic University S. Raffaele Roma, Milan, Italy; ^4^Institute for Macromolecular Studies, National Council of Research (CNR), Milan, Italy; ^5^IRCCS Fondazione Policlinico San Matteo, Pavia, Italy

## Abstract

**Objectives:**

Diabetic neuropathy is the most common complication of diabetes. The idea of alterations in energy metabolism in diabetes is emerging. The biogenic antioxidant R(+)-thioctic acid has been successfully used in the treatment of diabetic polyneuropathic (DPN) patients.

**Methods:**

The effects of R(+)-thioctic acid (1 tablet, 1.6 g) administration were evaluated in 12 DPN patients at baseline and at 15, 30, 60, and 120 administration days throughout the assessment of oxidative stress (OxS); ROS production rate by electron paramagnetic resonance (EPR) technique; and oxidative damage biomarkers (thiobarbituric acid reactive substances (TBARS) and protein carbonyls (PC)), electroneurography (ENG) and visual analogue scale.

**Results:**

Supplementation induced significant changes (*p* < 0.05) at 30 and 60 days. ROS production rate up to −16%; TBARS (−31%), PC (−38%), and TAC up to +48%. Motor nerve conduction velocity in SPE and ulnar nerves (+22% and +16%) and sensor conduction velocity in sural and median nerves (+22% and +5%). Patients reported a general wellness sensation improvement (+35%) at 30 days: lower limb pain sensation (−40%) and upper limbs (−23%).

**Conclusion:**

The results strongly indicate that an increased antioxidant capacity plays an important role in OxS, nerve conduction velocity, pain, and general wellness improvement. Nevertheless, the effects of the antioxidant compound were found positive up to 60 days. Then, a hormesis effect was observed. Novelty of the research would be a challenge for investigators to carefully address issues, including dose range factors, appropriate administration time, and targeting population to counteract possible “boomerang effects.” The great number of monitored parameters would firmly stress these conclusions.

## 1. Introduction

Type 2 diabetes mellitus (type 2 DM) is the most common form of diabetes, accounting for 90–95% of patients in developed countries: in 2030, up to 438 million people affected by type 2 DM have been estimated by recent studies [1].

Type 2 DM is a metabolic disorder, formerly known as non-insulin-dependent diabetes mellitus or adult-onset diabetes, resulting from a combination of insulin resistance and inadequate insulin secretion [2]. The basic metabolic disorder in type 2 DM can be identified in a gap in tissue oxygenation: this is a very important factor for the progression of microangiopathy. In fact, the idea of tissue-specific pathological alterations in energy metabolism in complication-prone tissues in diabetes is emerging [3].

Long-term and serious complications of diabetes develop gradually; they can eventually be disabling or even life-threatening [4]. One of the potential complications of diabetes includes nerve damage especially of the legs [5]. This condition, defined as diabetic peripheral neuropathy (DPN), is the most common complication of diabetes and is characterized by distal-to-proximal loss of peripheral nerve axons in about 30% of the patients [6, 7]. The involved neurons may be afferent (sensory), efferent (motor), or both [4]. Diabetic neuropathy develops on a background of hyperglycemia and associated metabolic imbalances, mainly oxidative stress (OxS). Hyperglycemia-induced overproduction of free radicals, in particular, reactive oxygen species (ROS), has been recognized as the source of further complications [8]. Recent evidence shows that OxS is central to the pathogenesis of DPN. The uppermost energy production and oxidation organelle of nerve cells, mitochondrion, have been proven to be the main place for oxidative stress production, playing an important role in the occurrence and development of type II diabetic peripheral neuropathy [9].

ROS promote apoptosis of vascular and neuronal cells and stimulate inflammation and pathological angiogenesis. However, imbalance occurs when free radical production exceeds the antioxidant capacity of patients with type II diabetes. ROS include free radicals, such as superoxide (O^2^•−), hydroxyl (HO•), peroxyl (RO^2^−•), and hydroperoxyl (HRO^2^−•^−^) and nonradical species, such as hydrogen peroxide (H_2_O_2_) and hydrochloric acid (HOCl) [10]. As it is known, they can be considered the main source of OxS and be responsible for damage to proteins, lipids, and DNA.

Electron paramagnetic resonance (EPR) is the only technique capable of providing direct evidence of the “instantaneous” presence of ROS leading to absolute concentration measurements [11, 12]. Indeed, short-lived ROS, if compared to the EPR time scale, are such EPR-invisible: only when “trapped” and transformed in more stable radical species, they become EPR detectable [13]. EPR spin trapping has therefore become an essential tool for ROS measurement in biological systems [14, 15].

DPN symptoms can be tingling, numbness, burning, or pain that usually begin at the tips of the toes or fingers and gradually spread upward. Poorly controlled blood sugar can eventually cause loose of feeling in the affected limbs [16].

Patients with painful sensory neuropathy due to diabetes might first complain of burning or itchy sensations or even pain in the feet and paresthesias [17]. Neuropathy frequently results in clinically significant morbidities, such as pain, loss of sensation, foot ulcers, and gangrene up to amputation. Painful DN deleteriously influences quality of life, sleep, mood, and the ability to work [16, 17]. Therefore, electroneurography (ENG) plays an important role in DN detecting, characterizing, and measuring.

Progression of diabetes mellitus is chronically accompanied by both sensory and motor neuropathies, with marked decrease in quality of life [18]. Sensory nerve degeneration has more conspicuous early signs, with slowing of nerve conduction velocity, clear signs of demyelination, and expressive symptoms related to somatosensory impairment [19]. Motor dysfunction is more subtle and considered as a late symptom in the pathology progression [20].

Nevertheless, the assessment of the severity of painful symptoms and nerve conduction slowing down is important not only to the diagnosis but also to the evaluation of treatment benefits.

In the previous studies, antioxidant administration, and in particular α-lipoic acid (ALA), has been demonstrated to be able to prevent the neurovascular abnormalities associated with diabetic neuropathy [21], to improve nerve blood flow and peripheral nerve fiber conduction, and to increase endoneurial glucose uptake and energy metabolism in DPN [22].

A short-term treatment—3-week duration of intravenous administration of ALA (600 mg/day)—was found to reduce the chief symptoms of diabetic polyneuropathy to a clinically meaningful degree [23], while an oral treatment for 4–7 months succeeded in reducing neuropathic deficits, favorable effects on neuropathic deficits without causing significant adverse reactions [24]. However, in a published trial [25], Dyck et al. [26] did not find an improvement in neurophysiology, quantitative sensory testing, and neuropathy scores. Moreover, the primary endpoint of that study did not change significantly in placebo-treated subjects and this brings into question the recurring dilemma of placebo effects in clinical trials on subjects affected by DPN [27]. To date, it is unclear whether the significant improvements observed after 3–5 weeks of oral administration at a dosage >600 mg/day are clinically relevant [28]. Thus, in a recent review article [4], ALA was described as a powerful antioxidant showing beneficial effects in subjects with DN [29]; nevertheless, by the news guidelines, there is not sufficient evidence to recommend it in DN treatment [30].

In NATHAN 1 trial study [25], 4 years of treatment with ALA in mild up to moderate asymptomatic DN patients resulted in a clinically meaningful improvement and prevention of progression of neuropathic impairments. Besides, studies aimed to confirm the effects of *α*-lipoic acid on glycemic control are lacking [31]. Indeed, despite the evidence of the clinical improvement of diabetic polyneuropathy, thanks to ALA supplementation reported in the literature, there are no studies showing changes in OxS during/after ALA administration for both short- and long-time periods.

In light of this, aim of the present study was to test short- and long-term effects, by oxidative stress and electroneurography measures, in type II diabetic patients with peripheral neuropathy, after supplementation of R(+)-thioctic acid, another antioxidant compound belonging to the lipoic acid family as well.

## 2. Materials and Methods

### 2.1. Subjects and Antioxidant Supplementation

According to the American Diabetes Association Standards Guidelines [32], the antioxidant activity of R(+)-thioctic acid (1 tablet, 1.6 g in a single morning dose for 120 days) in twelve (*n* = 12; 6 males and 6 females) diabetic patients also affected by peripheral neuropathy (DPN) was evaluated. In all patients, the diabetic neuropathy symptom scores were assessed to determine the presence of diabetic peripheral neuropathy. The same expert neurologist reasearcher examined all individuals. The diabetic neuropathy symptom (DNS) score was adopted. In short, it is a four-item validated symptom score, with a high predictive value to screen for polyneuropathy (PNP) in diabetes [33]. The score has the following items: (i) unsteadiness in walking, (ii) neuropathic pain, (iii) paraesthesia, and (iv) numbness in the legs or feet. Presence is scored 1, absence 0, and maximum score 4 points. A score of 1 or higher is defined as positive for PNP.

Subjects affected by hematologic diseases, hepatic, renal and/or heart failure, acute illness, chronic diseases like chronic infections, atherosclerotic diseases, alcohol abuse, smokers, and medication assumption that alter platelet functions were not included in the study.

Subjects' height and weight measurements, blood pressure, SaO_2_, heart rate (HR), age, and accompanying disease history were determined. Body mass index (BMI) was calculated according to the Quetelet index, based on the (WT·(HT)^−2^) formula. All subjects' characteristics are reported in Table 1. All subjects underwent clinical examination for diabetes and neuropathy to confirm the indication for R(+)-thioctic acid supplementation. They were studied in the morning, after 8 a.m.

### 2.2. Experimental Design

Each subject reached the clinical department at 8 : 00 a.m., overnight fasting for blood test. Subjects were fully informed of the procedure and the involved risks and, before data collection, signed a written informed consent outlining study requirements. The procedure was conducted according to the Declaration of Helsinki, and approval was obtained from the institutional Ethics Committee of the Istituto Auxologico Italiano, Milan, Italy (N° 23 C 209 - DIAB EPR).

**Figure 1:**
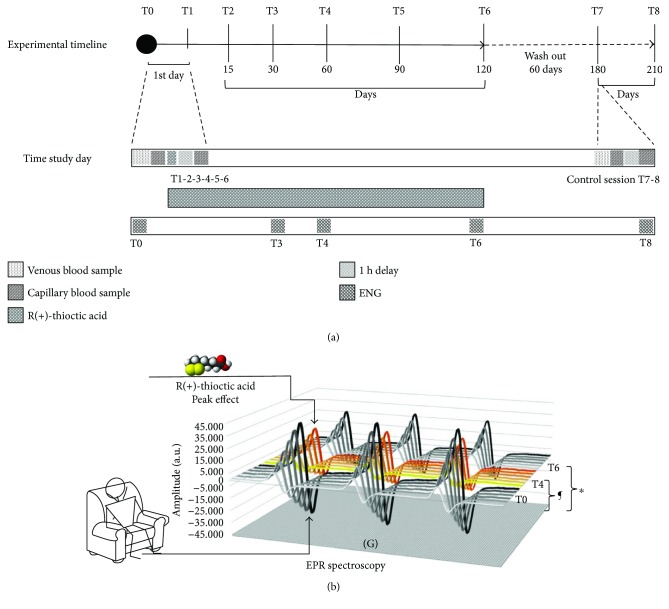
Scheme of the experimental protocol. Experimental timeline of the protocol adopted to monitor the effects of R(+)-thioctic acid supplementation on glucose levels, hematological profile, ROS production rate, oxidative damage, and ENG in type II diabetic neuropathic patients. The three monitored periods, study session (120 days: T0–T6), wash out (60 days: T6-T7), control session (30 days: T7-T8), are indicated (a). Sketch of the experimental protocol adopted to measure the EPR determined ROS production rate. The stacked plots of the EPR spectra recorded at the baseline (T0), 60 days (T4, yellow spectra), and 120 days (T6) are displayed. The significant differences ^∗^*P* < 0.05 and ^¶^*P* < 0.0001 are indicated (b).

The experimental protocol adopted to monitor oxidative stress, biochemical, and hematologic evaluations of DPN subjects is summarized in the sketch displayed in Figure 1. In particular, to assess ROS production, for the baseline evaluation (T0), the subject remained seated for 30 minutes before the ROS production measurement on capillary blood by adopting the microinvasive method previously developed by some of us [12, 34, 35]. The ROS production rate in capillary blood was also tested one hour after the administration of a tablet of R(+)-thioctic acid (1 cp, 1.6 g) (T1). Then, the subjects were daily treated with R(+)-thioctic acid (1 cp, 1.6 g) for 120 days.

Capillary blood was drawn from the fingertip at 15 (T2), 30 (T3), 60 (T4), 90 (T5), and 120 (T6) days. At that time, the supplementation was terminated. 60 days after the end of supplementation (wash out), the patients were tested again in the department and then monitored for other two months (control session), that is, at 180 (T7) and 210 (T8) days without antioxidant administration.

### 2.3. Blood Sample Collection

For each subject, venous blood (≈10 mL) was drawn from an antecubital vein and collected in a heparinized vacutainer tube (Becton Dickinson and Company, UK) at T0, T2, T3, T4, T5, T6, T7, and T8 for oxidative stress (OxS) determination. Plasma was separated by centrifuge (5702R, Eppendorf, Germany) at 3000*g* for 5 min at 4°C and immediately stored in multiple aliquots at −80°C until being assayed. Samples were thawed only once before analyses, performed within two weeks from collection.

Furthermore, at T0, T3, T4, T6, and T8, venous blood samples were drawn for the standardized clinical hematological analyses. Parameters were determined by using an automated hematology analyzer, according to the standard analysis methods of Istituto Auxologico S. Luca laboratories.

### 2.4. Biochemical and Hematologic Parameters

The evaluated biochemical and hematologic parameters were hemoglobin A1c (HbA1c %); glycaemic index (GI); erythrocyte indices—red blood cell count (RBCs), haemoglobin (HGB), hematocrit (HCT), mean corpuscular volume (MCV), mean corpuscular hemoglobin (MCH), mean corpuscular hemoglobin concentration (MCHC), and red cell distribution width (RDW); leukocyte indices—white blood cells (WBCs), lymphocyte (LYM), monocytes (MON), and granulocytes (GRA); platelet indices—platelets (PLT), mean platelet volume (MPV), S-lactate dehydrogenase (LDH), S-creatinine, and C-reactive protein (CRP).

### 2.5. Enzymatic Parameters Determination

All plasma samples were assessed by immunologic and/or enzymatic methods by using a microplate reader spectrophotometer (Infinite M200, Tecan, Austria). All measurements were performed in duplicate, and the interassay coefficients of variation were found in the range indicated by the manufacturer.

#### 2.5.1. Antioxidant Capacity

Plasma total antioxidant capacity (TAC) was measured by an enzymatic kit (Cayman Chemical, U.S.). This assay is based on the ability of the antioxidants present in plasma to inhibit the oxidation of 2,2′-azino-bis(3-ethylbenzothiazoline-6-sulfonic acid) (ABTS) to the radical cation (ABTS^+^) by a peroxidase. The amount of produced ABTS^+^ is then determined by measuring the absorbance signal at 750 nm. The antioxidant concentration is proportional to the absorbance signal suppression. TAC concentration was calculated from a trolox (6-hydroxy-2,5,7,8-tetramethylchroman-2-carboxylic acid) standard curve and expressed as trolox equivalent antioxidant capacity concentration (mM).

#### 2.5.2. Thiobarbituric Acid Reactive Substances (TBARS)

The herein adopted TBARS assay kit (Cayman Chemical, USA) allowed a rapid photometric detection of the thiobarbituric acid malondialdehyde (TBAMDA) adduct at 532 nm. A linear calibration curve was built up from pure MDA-containing solutions.

#### 2.5.3. Protein Carbonyls (PC)

Reactive species directly or indirectly produced by lipid peroxidation intermediates may also produce an oxidative protein modification that in turn can be measured by the reactive carbonyl content. A protein carbonyl assay kit (Cayman Chemical, USA) was used to colorimetrically evaluate oxidized protein concentration. The samples were spectrophotometrically read at 370 nm, as described in detail by the manufacturer. The calculated values were normalized to the total protein concentration in the final pellet (absorbance at 280 nm), in order to take into account the protein loss during the washing steps, as suggested by the kit's user manual. All samples were determined in duplicate, and the interassay coefficients of variation resulted in the range indicated by the manufacturer.

### 2.6. EPR Protocol for ROS Detection in Blood and Plasma

EPR experiments were carried out by means of an e-scan EPR spectrometer (Bruker, Germany) operating at the common X-band microwave frequency (~9.8 GHz). The instrument was interfaced to a temperature and gas controller Bio III unit (Noxigen Science Transfer & Diagnostics GmbH, Germany). ROS production rate was determined [12, 13, 34] using capillary blood samples immediately treated with CMH solution (1 : 1). 50 *μ*l of the obtained solution was put in a glass EPR capillary tube (Noxygen Science Transfer & Diagnostics, Germany) placed inside the instrument cavity for data acquisition.

Plasma samples separated by centrifuge (from venous blood) were also used to measure ROS formation by EPR as well. ROS production rate was determined at rest by means of an EPR method recently implemented on plasma samples [35].

All spectra were collected by adopting the same acquisition parameters and handled by using the software standardly supplied by Bruker (Win EPR System, V. 2.11). Acquisition parameters were microwave frequency: 9.652 GHz; modulation frequency: 86 kHz; modulation amplitude: 2.28 G; sweep width: 60 G; microwave power: 21.90 mW; number of scans: 10; and receiver gain: 3.17^.^10^1^. Sample temperature was firstly stabilized and then kept at 37°C by the temperature controller unit. The collected data allowed us to attain a relative quantitative determination of the sample ROS production rate. The data were, in turn, converted in absolute concentration values (*μ*mol·min^−1^) by adopting the CP^•^ (3-carboxy-2,2,5,5-tetramethyl-1-pyrrolidinyloxy) stable radical as external reference.

### 2.7. Assessment of Peripheral Nerve Function: Electroneurography (ENG)

Electroneurography (ENG) parameters were obtained by using a Neuropack MEB9400 (Nihon Kohden Corporation, Japan) keypoint device able to record and collect the ENG signals. The temperature of the room was kept constant at 21°C.

Motor nerve conduction velocity (MNCV) of ulnar and median nerves and sensory nerve conduction velocity of popliteal and sural nerves (SNCV) were measured using surface electrodes (AMBU Neuroline, USA).

### 2.8. Visual Analog Scale (VAS)

Subjective mood, general wellness (happy/unhappy, rested/tired, and welfare/malaise), and pain (no pain/upper limbs pain, no pain/lower limbs pain, and no pain/foot-feet pain) were evaluated using a 0–100 mm visual analog scale (VAS) to test the subjective perception of the antioxidant supplementation effects. Patients with VAS pain scores of 30 mm or less were classified as affected by mild pain. Those with scores of 70 mm or more were considered affected by severe pain, while scores from 31 to 69 mm were ascribed to moderate pain. The classification was based, in part, on the findings of Collins et al. [36]. The patients repeated the VAS measurement at every session, from the baseline (T0) until the end of the evaluation period (T8).

### 2.9. Statistical Analysis

All data were presented as mean ± SD and were analysed using repeated Shapiro-Wilks *W* test. A normal distribution was adopted as the null hypothesis that was rejected if the critical value *P* for the statistic *W* test resulted less than 0.05. Experimental data were compared by ANOVA variance analysis followed by Bonferroni's multiple comparison test to further check the among group significance (GraphPad Prism 7, Software Inc., San Diego, CA). Pearson's product moment correlation coefficient (*R*) with 90% confidence intervals (CI) was used to show up possible relationships between selected parameters. *P* < 0.05 statistical significance level was accepted. Change ∆% estimation [((post value-pre value)/pre value)^.^100] is also reported in the text. Sample size calculation to determine the minimum number of subjects for adequate study power was made by using the Freeware G^∗^Power software (http://www.psycho.uni-duesseldorf.de/abteilungen/aap/gpower3/). At 80% power, the calculated sample size was of 11 subjects, slightly lower than the subject's population recruited in the present study.

## 3. Results

### 3.1. R(+)-Thioctic Acid Effects on Anthropometric and Haematologic Parameters

During the supplementation period of R(+)-thioctic acid and for the whole duration of the study, subjects' weight, BMI, SaO_2_, blood pressure, heart rate, and pharmacological therapy did not show any significant difference. At T0, the biochemical parameters and the haematological indices resulted in the reference ranges as follows: erythrocyte indices: RBCs: 4.63 ± 0.48 10^6^/mm^3^, HGB: 14.04 ± 1.16 g/dL, HCT: 41.46 ± 3.52%, MCV: 89.83 ± 2.60 *μ*m^3^, MCH: 30.44 ± 1.04 pg, MCHC: 33.89 ± 0.47 g/dL, and RDW: 13.78 ± 0.95%; leukocyte indices: WBCs: 6.58 ± 1.60 10^3^/mm^3^, %LYM 27.65 ± 6.87, %MON 8.95 ± 2.62, %GRA 58.94 ± 8.88, #LYM: 1.89 ± 0.83 10^3^/mm^3^, #MON: 0.56 ± 0.10 10^3^/mm^3^, and #GRA 3.81 ± 0.80 10^3^/mm^3^; platelet indices: PLT: 219.75 ± 51.01 10^3^/mm^3^ and MPV: 9.50 ± 0.92 *μ*m^3^; S-lactate dehydrogenase ((LDH): 327.63 ± 77.55 U/L); S-creatinine (0.86 ± 0.32 *μ*mol/L); C-reactive protein ((CRP): 0.23 ± 0.33 mg/L). By contrast, haemoglobin A1c ((HbA1c): 6.93 ± 0.69%) and glycaemic index ((GI): 165.77 ± 32.34 mg/dL) resulted strong increases with respect to the normal range.

All biochemical and haematological parameters, monitored at T3, T4, T6, and T8, did not show any statistically significant differences with respect to the basal T0 level.

However, the glycaemic index followed a trend parallel to OxS, showing an 8% decrease at T4 and a 26% increase at T6, slowly returning to the basal level after the wash out time period.

### 3.2. R(+)-Thioctic Acid Effects on ROS Production and Oxidative Damage Biomarkers

**Figure 2:**
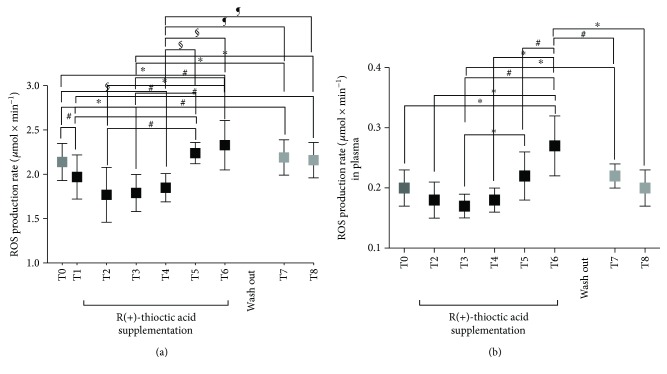
Time course of the ROS production rate (*μ*mol·min^−1^) in capillary blood (a) and plasma (b) calculated by the EPR-recorded spectra at the protocol times (T0–T8). Data are expressed as mean ± SD. Times without supplementation (T0, T7, and T8—grey full squares); times following antioxidant (R(+)-thioctic acid) supplementation (black full squares). ^∗^*P* < 0.05, ^#^*P* < 0.01, and ^§^*P* < 0.001, significant differences. ^¶^*P* < 0.0001.

The antioxidant supplementation of R(+)-thioctic acid induced EPR detectable changes in the ROS production levels (*μ*mol·min^−1^) measured at the different times (T0–T8) in type II DN patients' capillary blood and plasma. The ROS production rate data calculated at any time and the statistically significant differences between times of measurements are shown in Figures 2(a) and 2(b) in capillary blood and plasma, respectively. More specifically, the differences between brackets in the Figure are as follows:

- Capillary blood (A):
- T0 (2.14 ± 0.21) versus T1 (1.97 ± 0.25), T2 (1.77 ± 0.31), T3 (1.79 ± 0.21), and T6 (2.33 ± 0.28); T1 (1.97 ± 0.25) versus T6 (2.33 ± 0.28), T7 (2.19 ± 0.20), and T8 (2.16 ± 0.20); T2 (1.77 ± 0.31) versus T5 (2.24 ± 0.12) and T6 (2.33 ± 0.28); T3 (1.79 ± 0.21) versus T5 (2.24 ± 0.12), T6 (2.33 ± 0.28), T7 (2.19 ± 0.20), and T8 (2.16 ± 0.20).
- in Plasma (B):
- T0 (0.20 ± 0.03) versus T6 (0.27 ± 0.04); T2 (0.19 ± 0.03) versus T6 (0.27 ± 0.04); T3 (0.18 ± 0.03) versus T5 (0.22 ± 0.02), T6 (0.27 ± 0.04), and T7 (0.22 ± 0.02); T4 (0.22 ± 0.02) versus T6 (0.27 ± 0.04); T5 (0.22 ± 0.02) versus T6 (0.27 ± 0.04); T6 (0.27 ± 0.04) versus T7 (0.22 ± 0.02) and T8 (0.21 ± 0.02).

**Figure 3:**
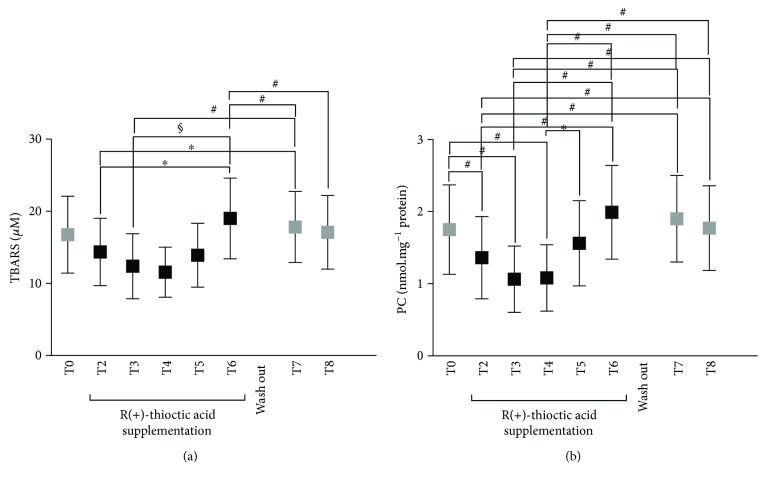
Time course of TBARS (mM) (a) and PC (nmol·mg^−1^ protein) (b) concentration data at the protocol times (T0–T8). Data are expressed as mean ± SD. Times without supplementation (T0, T7, and T8—grey full squares); times following antioxidant (R(+)-thioctic acid) supplementation (full squares). ^∗^*P* < 0.05, ^#^*P* < 0.01, and ^§^*P* < 0.001, significant differences.

At the same time, the antioxidant supplementation induced changes in the investigated oxidative markers. TBARS (*μ*M) and PC (nmol·mg^−1^ protein) concentration data measured at any time and the statistically significant differences between times of measurements are shown in Figures 3(a) and 3(b), respectively.

More specifically, the differences between brackets in the Figure are as follows:

- TBARS (A):
- T2 (14.37 ± 4.68) versus T6 (19.02 ± 5.60) and T7 (17.82 ± 4.93); T3 (12.38 ± 4.51) versus T6 (19.02 ± 5.60) and T7 (17.82 ± 4.93); T4 (11.57 ± 3.49) versus T6 (19.02 ± 5.60) and T7 (17.82 ± 4.93).
- PC (B):
- T0 (1.75 ± 0.62) versus T2 (1.39 ± 0.60), T3 (1.63 ± 0.46), T4 (1.08 ± 0.46), and T5 (1.56 ± 0.59); T2 (1.39 ± 0.60) versus T6 (1.99 ± 0.65), T7 (1.90 ± 0.60), and T8 (1.77 ± 0.59); T3 (1.63 ± 0.46) versus T6 (1.99 ± 0.65), T7 (1.90 ± 0.60), and T8 (1.77 ± 0.59); T4 (1.08 ± 0.46) versus T5 (1.56 ± 0.59), T6 (1.99 ± 0.65), T7 (1.90 ± 0.60), and T8 (1.77 ± 0.59); T5 (1.56 ± 0.59) versus T6 (1.99 ± 0.65).

**Figure 4:**
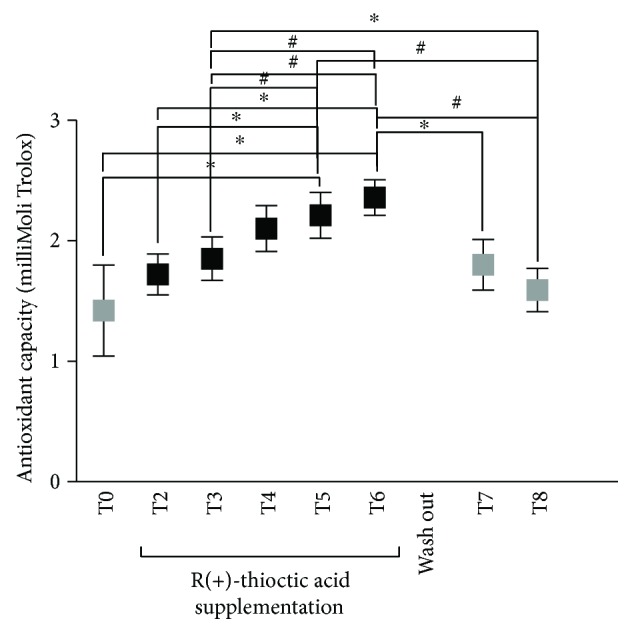
Time course of the antioxidant capacity (TAC mMol Trolox) concentration data in plasma at the protocol times (T0–T8). Data are expressed as mean ± SD. Times without supplementation (T0, T7, and T8—grey full squares); supplementation times (T2–T6—full squares). ^∗^*P* < 0.05, ^#^*P* < 0.01 significant differences.

Finally, the antioxidant capacity in plasma, TAC (mM) concentration data measured at any time, and the statistically significant differences between times of measurements are reported in Figure 4.

More specifically, the differences between brackets in the Figure are as follows:

T0 (1.42 ± 0.38) versus T5 (2.21 ± 0.19) and T6 (2.36 ± 0.15); T2 (1.72 ± 0.17) versus T5 (2.21 ± 0.19) and T6 (2.36 ± 0.15); T3 (1.85 ± 0.18) versus T5 (2.21 ± 0.19) and T6 (2.36 ± 0.15); T4 (2.10 ± 0.19) versus T6 (2.36 ± 0.15) and T8 (1.59 ± 0.18); T5 (2.21 ± 0.19) versus T8 (1.59 ± 0.18); T6 (2.36 ± 0.15) versus T7 (1.80 ± 0.21) and T8 (1.59 ± 0.18).

### 3.3. R(+)-Thioctic Acid Effects on Electroneurography (ENG)

In the patient group examined in the present study, the four item symptom DNS score resulted as follows: neuropathic pain: 100% (12 patients); paraesthesia: 58% (7 patients); unsteadiness in walking: 8% (1 pz); numbness in the legs or feet: 0%. Therefore, a score (±SD) of 1.6 ± 0.65, positive for PNP, was calculated. ENG abnormalities were monitored in all patients. Changes in signal amplitude (mV) and distal latency (m/s) in sensory and motor nerves at T0, T3, T4, T6, and T8 were observed (data not shown).

**Figure 5:**
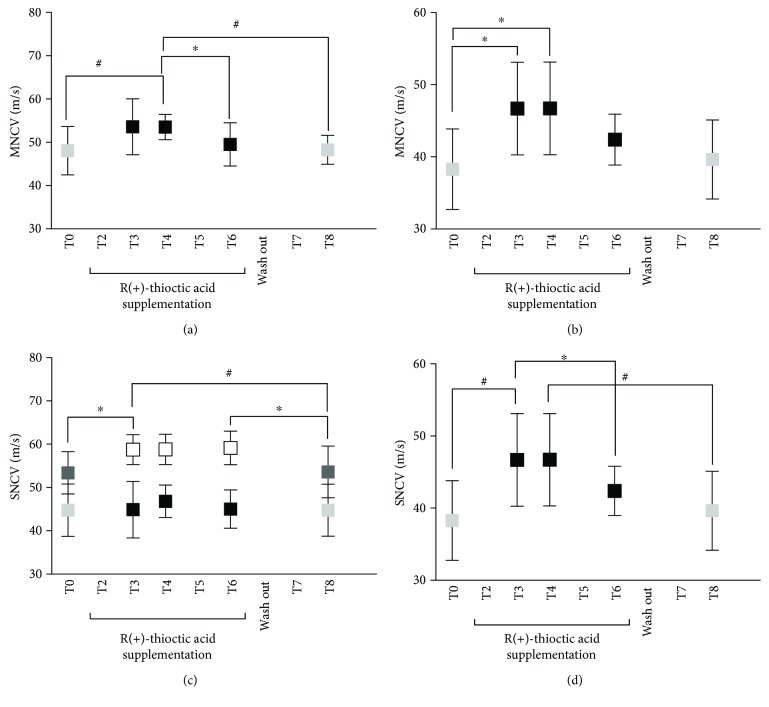
Time course of motor nerve conduction velocity ((MNCV) m/s) data measured in the ulnar (a) and SPE (b) nerves. At the times without supplementation (T0 and T8—grey full squares); during the supplementation period (T3–T6—full squares). Sensory nerve conduction velocity ((SNCV) m/s) data measured in median wrist (full square symbols) and wrist-elbow (empty square symbols). Data at times without supplementation (T0 and T8—light and dark full grey squares, resp.) (c) and sural nerves (full and grey squares as well) (d). Data are expressed as mean ± SD. ^∗^*P* < 0.05 and ^#^*P* < 0.01, significant differences.

R(+)-thioctic acid supplementation induced changes in motor (MNCV m/s) and sensitive (SNCV m/s) conduction velocities (see Figure 5) calculated from the collected signals between different time points. More specifically, the significant differences between different time points, displayed between a bracket in the Figure, resulted as follows:

MNCV (m/s) in ulnar nerve increased significantly at (Figure 5(a)):

T3 (53.62 ± 6.48) versus T0 (48.06 ± 5.58); T3 versus T6 (49.52 ± 4.98) and T8 (48.28 ± 5.36);

superficial peroneal nerve (SPE) MNCV (m/s) increased significantly at (Figure 5(b)):

T3 (46.68 ± 6.42) and T4 (46.70 ± 6.43) versus T0 (38.30 ± 5.53).

SNCV (m/s) in median wrist-elbow changed significantly at (Figure 5(c)):

T0 (52.38 ± 4.89) versus T3 (58.76 ± 3.24); T3 (58.76 ± 3.24) versus T6 (59.16 ± 3.90) and T8 (53.60 ± 5.96); T6 (59.16 ± 3.90) versus T8 (53.60 ± 5.96).

SNCV (m/s) in sural nerve changed significantly (Figure 5(d)) at T0 (38.29 ± 5.54) versus T3 (46.68 ± 6.42); T3 (46.7 ± 6.42) versus T6 (42.40 ± 3.44) and T8 (39.63 ± 5.48).

### 3.4. Visual Analog Scale (VAS)

**Figure 6:**
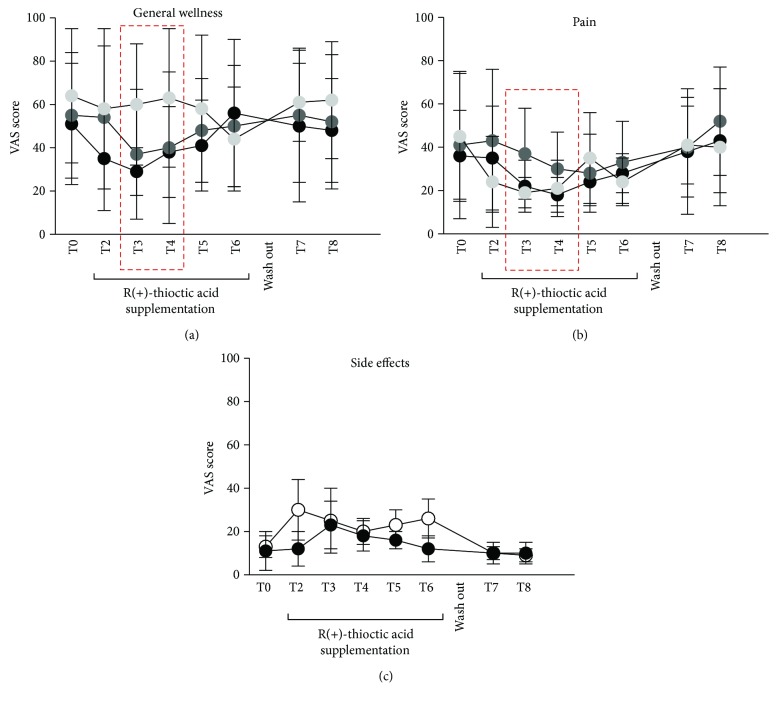
VAS assessment for general wellness item (a) happy/unhappy (light grey circle), rested/tired (grey circle), and welfare/malaise (full circle); Pain item (b) no pain/pain upper limbs (open circle), no pain/pain lower limbs (grey circle), and no pain/pain foot-feet (full circle); and side effects (c) stomachache (open circle) and nausea (full circle) from T0 to T8. The time period correspondent to the measured best positive effects of the treatment on general wellness and pain sensation is squared in red.

Patients reported an improved sense of well-being (Figure 6(a)), particularly in the welfare/malaise item after 30–60 days of antioxidant supplementation (T3-T4) (VAS percentage change of about +35%). Also, pain sensation decreased (Figure 6(b)) of about the 50% in upper limbs, the 23% in lower limbs, and the 40% in feet. However, side effects of R(+)-thioctic acid, particularly after long supplementation periods, were referred (Figure 6(c)) as stomachache at T2 (+130%) and nausea at T3 (+110%).

### 3.5. Correlations between Parameters

**Figure 7:**
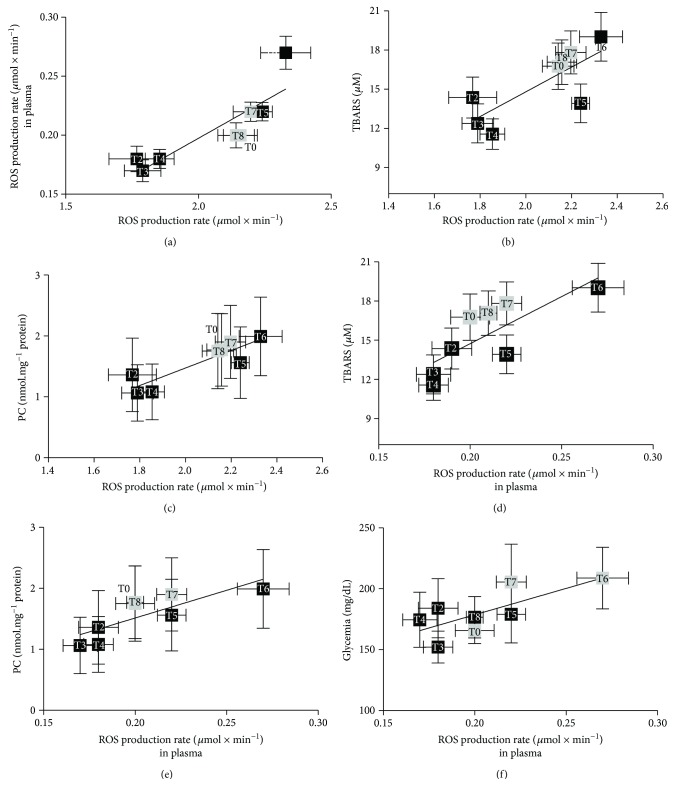
Panel plots (for better graphical visualization, the data are reported as mean ± standard error of mean (SEM)) of the ROS production rate data in capillary blood (a, b, c) and plasma (d, e, f) versus the biomarkers of oxidative damage (TBARS and PC) and the glycaemic values. The linear regression lines are reported in each panel showing very strong correlation between the EPR data and the other variables (see text). The time of the measurement (as T0–T8) is reported inside each data point.

A positive linear relationship was found at each supplementation time between the EPR data, and both oxidative damage biomarkers and glycaemic index data shown in the panel plots of Figure 7 where the ROS production rate in capillary blood data versus the ROS production rate values calculated in plasma (a), TBARS (b), and PC (c) as well as the ROS production rate calculated in plasma versus TBARS (d), PC (e), and glycemic values (d) and the linear regression lines are displayed.

In particular, ROS production in capillary blood versus ROS in plasma (*R*^2^ = 0.76, *p* < 0.01) (a), TBARS (*R*^2^ = 0.60, *p* < 0.01) (b), and PC concentration data (*R*^2^ = 0.79, *p* < 0.01) (c) and ROS production in plasma versus TBARS (*R*^2^ = 0.58, *p* < 0.01) (d), PC (*R*^2^ = 0.66, *p* < 0.01) (e), and glycaemic values (*R*^2^ = 0.54, *p* < 0.05) (f) are also displayed.

## 4. Discussion

As widely reported, in a number of various diseases, including type 2 DM, excessive ROS levels as well as decreased antioxidant defenses cause oxidative stress (OxS). In particular, multiple sources of OxS in diabetes can be found, including nonenzymatic (i.e., glycation process), enzymatic (i.e., glucose autooxidation), and mitochondrial pathways [37, 38]. Hyperglycemia-induced oxidative stress activates programmed neuron death, which contributes to the onset of the diabetic neuropathy pathology [39, 40].

Dietary supplementation with *α*-lipoic acid, evening primrose oil (EPO), or sunflower oil was found to decrease plasma lipid levels and risk factors [41]. The positive effects of *α*-lipoic acid on oxidative stress parameters are reported in the literature: increased super oxide dismutase (SOD) and subsequent reduction of H_2_O_2_ by catalase [31, 42].

Direct evidence of OxS in diabetes was previously provided by the measurement of oxidative stress markers, such as plasma and urinary F2-isoprostane, as well as plasma and tissue nitrotyrosine and superoxide anion radicals (O•^2−^) levels [43, 44].

Starting from these considerations, the present study aimed at investigating the effects produced by antioxidant supplementation (four months, R(+)-thioctic acid, 1.6 g/die) and after the wash out period (two months) on diabetic neuropathic patients throughout ROS production measurement by adopting an innovative EPR method.

### 4.1. Oxidative Stress

The study protocol was sketched in Figure 1 where the steps followed to determine the ROS production levels by EPR technique are reported. The method, built up by some of us, was previously demonstrated to return reliable absolute concentration values in a simple way thanks to the adopted noninvasive procedure by using fingertip capillary blood [12, 34, 35]. In healthy subjects, the positive correlation of the EPR capillary blood data with respect to plasma as well as biomarker damage (TBARS and PC) parameters was also demonstrated [13]. However, the protocol of the present study also provided to collect venous blood samples to determine the haematological parameters. Therefore, the EPR data could be additionally recorded on plasma samples, and the correlation of blood capillary and plasma EPR data, as well as oxidative biomarkers, could be investigated (see Figure 7 and below in the text).

As observed in Figure 2, in all patients, antioxidant supply induced significant EPR detectable changes in ROS formation. The kinetics of ROS production was estimated at different time periods from the signal intensities of the EPR-collected spectra, in capillary blood (Figure 2(a)) and plasma (Figure 2(b)), respectively, and at the baseline (T0), one hour after (T1) and during the entire period of treatment with R(+)-thioctic acid (T2, T3, T4, T4, and T6) as well as during the wash out period (T7 and T8).

As a matter of fact, in all patients at T0 that is just before the antioxidant supply (basal level), a high degree of oxidative damage, as confirmed by the high ROS production rate concentration values, was measured in both capillary blood and plasma.

Indeed, the acute effects, “one shot” of R(+)-thioctic acid supplementation in differently aged healthy subjects, were previously reported [34]. A decrease of the ROS production rate level, subsequent to the acute supply (1 cp, 1.6 g) between 60 and 90 minutes (about −2% and 4%, resp.), was reported.

Indeed, the results of the present study conducted on diabetic patients, homogeneous for age and physical characteristics (see Table 1) and concerning a prolonged R(+)-thioctic acid supply, evidenced two opposite, beneficial as well as adverse, effects mainly depending on the supplementation duration.

Focusing on the beneficial ones, crucial importance played the evidence of the significant OxS decrease. In capillary blood, ROS production significantly decreased at T1 (1 h, −8%, *p* < 0.01) and kept decreasing at T3 (30 d, −16%, *p* < 0.01) and T4 (60 d, −14%, *p* < 0.0001) of daily supplementation. According to the ROS decrease, also, TBARS and PC levels significantly (*p* < 0.01) decreased at T3 (−26 and −39%) and T4 (−31% and 38%), respectively (Figures 2 and 3).

The results of the present study were found in agreement with previously reported data, where the TBARS increasing trend, observed in diabetic subjects, was found reversed by a combination with C and E vitamins and *β*-carotene treatment, once that the stabilization of the diabetes pathology was ensured [45].

On the contrary, in the present study, the adverse effects showed up by the data collected at T5 and T6 (90–120 d): the OxS values significantly (*p* < 0.01) increased as shown by the increase of ROS production in capillary blood (+5 and +9%, resp.) and plasma (+10, +35%), when compared to the basal level (T0, see Figure 2). At the same protocol times, the oxidative damage markers, TBARS and PC, were found significantly (*p* < 0.01) increased (+13 and +14%, see Figure 3) too.

Type 2 diabetic patients are exposed, among others, to an increase of lipid peroxidation; hyperglycemia can induce OxS via glucose oxidation, nonenzymatic glycation of proteins, disruption of the polyol pathway, altered eicosanoid metabolism, and decreased antioxidant defences [46, 47]. As it is known, antioxidants participate in the mechanisms for reducing the deleterious free radical effects by preventing their production and/or inactivating them through enzymatic defence systems [47].

The very low antioxidant status level in patients affected by diabetes type 2, complicated by peripheral neuropathy, previously reported [4], was confirmed by the present study too.

An increase of the antioxidant capacity was observed for the entire supplementation duration (Figure 4). Concentrations were kept high with respect to the basal value (T0), reaching the highest level at the end of the administration time T6 (120 d, +66%, *p* < 0.05), then returning slowly towards the baseline during the wash out and the periods without administration even if at a slightly higher level (T8, +12%). This figure was found also for the ROS production and oxidative damage markers (see Figures 2 and 3, T8).

The observation of a biphasic adaptive response to antioxidant supplementation, as shown in Figure 4, could be regarded as a low-dose hormetic stimulation, accompanied by a high-dose hormetic inhibition [48]. Indeed, hormesis has recently become a key model in toxicology and pharmacology [48–50]. U-shaped dose-response mechanisms have been recognized by researchers to be involved in factors affecting memory, learning, and performance, as well as dietary antioxidant supplements and/or oxidative stress-mediated degenerative reactions [48]. Under this evidence, particular attention has been paid to the neuroprotective properties of antioxidants (i.e., polyphenols) [49]. In well agreement to the hormesis concept, some substances (i.e., drugs, toxins, and natural substances) administered at low dose may elicit a positive response in terms of adaptation to or protection from the stressor, whereas at higher concentrations, the toxic effect will prevail [51–53]. For example, flavonoids and other polyphenolic compounds have been reported to play powerful antioxidant effects in many in vitro test systems, whereas acting as prooxidants at higher concentrations [54].

In the present study, the loose of the benefits observed between T4 and T6 supplementation times seems to confirm a prooxidant effect played by R(+)-thioctic acid on ROS production and oxidative damage markers, as well as on motor and sensory nerve conduction velocities (see Figure 5 and the text below). A hormetic effect seems to be suggested also by the observed decrease in ROS production and oxidative damage biomarker levels after two months of wash out (T7 and T8, see Figures 2 and 3). This finding indicated the return of the antioxidant capacity, continuously increasing during the administration times, to lowering levels, almost corresponding to those recorded under basal conditions (T0) (see Figure 4).

### 4.2. Electroneurography

The role played by OxS in nerve damage has been extensively studied in experiments carried out in an animal model [55] and more specifically in diabetic human subjects [56].

Diabetic neuropathies are diabetes mellitus-associated disorders probably ascribable to different causes; some of them are not yet well elucidated and in particular how prolonged exposure to high blood glucose levels can produce nerve damage.

Motor nerve (MNCV) and sensory nerve (SNCV) conduction velocities resulted in the primary endpoints for studying the therapeutic effectiveness of *α*-lipoic acid on nerve functions. In fact, *α*-lipoic acid has been shown to improve motor nerve conduction velocity in experimental diabetic neuropathy and to protect peripheral nerves from ischemia in rats [57].

Evidence related to changes in nerve conduction velocity after thioctic acid administration was observed primarily in experimental models [58–60].

The conduction velocities (m/s) of the sensory component of ulnar and sural nerves in diabetic subjects affected with neuropathy were previously found significantly lower than those measured in diabetics without neuropathy as well as nondiabetic subjects [61]. In well agreement with the literature data [62, 63], in the present study, a reduction of the motor and sensory conduction velocities was measured at the baseline (T0, see Figure 5).

Again, the literature data [63] reported an increase of both MNCV and SNCV as well as an improvement of the neuropathy signs in patients treated with *α*-lipoic acid (600 mg/die). This molecule is known to interact with many glucose-converting enzymes, fatty acids, and other energy sources in ATP. In the present study, nerve conduction significantly increased at T3 and T4 (30–60 supplementation days): MNCV: +16-17% in ulnar and external sciatic-popliteal (SPE) nerves; SNCV: +12-13% in median and about +16% in sural nerves.

Indeed, in human studies to date, a great number of studies targeting “thioctic acid” and “diabetes mellitus” have been published, from which only a few randomized control trials in humans are distinguished in terms of DPN treatment with thioctic acid. We are well aware that nerve conduction velocity cannot be used to monitor changes in oxidative stress; nonetheless, the data of the present study suggested us that the hormesis effect described above could be hypothesized for MNCV and SNCV too: the beneficial effects on nerve conduction were lost after long antioxidant administration time period (T6 and over), almost returning to the pretreatment (T0) conduction velocity values (see Figure 5).

### 4.3. Visual Analog Scale

VAS is a suitable method to measure pain/pain relief and general wellness. In the present study, in all patients, a regression of the subjective pain sensation as well as a general wellness improvement was reported under R(+)-thioctic acid treatment mainly at T3 and T4 (see Figures 6(a) and 6(b)).

It is true that “pain is a private-personal experience and it's impossible for us to know precisely what someone else's pain feels like” [64]. However, even if this is not an objective status, because pain results from complex processes involving neurophysiological and psychological mechanisms, subjective sensations are important to evaluate the quality of life.

Mild pain was referred at the baseline (45 ± 29 mm), followed by an improvement of about the 50% after 30–60 days of treatment (T3-T4; 19 ± 7 and 21 ± 13 mm, resp.), then returning to the basal level after the wash out period. At the same time, at T3-T4, the general wellness sensation was found to improve at about +35% as well.

### 4.4. Side Effects

Side effects, like stomachache and nausea, were reported, but mild or moderate, since no patients interrupted the supplementation for this reason.

Side effects as a consequence of alpha-lipoic acid oral administration were previously reported by Ziegler et al. [65]. Despite the low-dose protocol (600 mg/die) adopted by the authors, patients reported mild nausea, vomiting, and vertigo.

A significantly higher dose protocol was followed in the present study (1.6 g/die): patients referred stomachache and nausea mainly at T2-T3 and T5-T6, the symptoms returning to normal after the wash out period (see Figure 6(c)).

Nevertheless, during the supplementation time course, patients reported an improvement of the general wellness sensation mainly at T3-T4: in particular, pain at the lower limbs and feet (see Figures 6(a) and 6(b)) was felt to really decrease. This beneficial subjective sensation ended at T5 and T6 (90 and 120 d) with the return to the basal score or even to a pain increase.

### 4.5. Hematologic Parameters

Despite the high dose and the long-time (four months) duration of the treatment, no significant differences were found in the haematological parameters all over the supplementation duration.

### 4.6. Data Correlations

As already pointed out, OxS plays an important role in type II DPN. Thus, possible correlations among all the parameters measured in this study were investigated and the results are displayed in Figure 7. A significant (*p* < 0.01) linear correlation was found at every time between the ROS production mean values in capillary blood and ROS production in plasma (*R*^2^ = 0.76), TBARS (*R*^2^ = 0.60), and PC (*R*^2^ = 0.79) (see Figures 7(a)–7(c)). These findings allowed us to extend the results previously obtained in healthy subjects, confirming a correlation of OxS and all related parameters [12]. Moreover, significant (*p* < 0.01–*p* < 0.05) relationships were found at every time between ROS production mean values in plasma and TBARS (*R*^2^ = 0.58), PC (*R*^2^ = 0.66), and glycaemic levels (*R*^2^ = 0.54) (see Figures 7(d)–7(f)), so confirming the peculiar role played by OxS, measured by both ROS production and oxidative damage in diabetes pathology.

### 4.7. Limits of the Study

The authors are aware that the current study suffers from certain limitations. Among them, the most relevant is the number of participants in the study. Indeed, the sample size (*n* = 12; 6 male and 6 female diabetic patients) was large enough to ensure an adequate study power, as determined by the Freeware G^∗^Power software (see paragraph 2.7). In fact, as already pointed out, at 80% power, the calculated sample size was 11 subjects. However, the population recruited for the study is certainly well below the number of participants standardly recruited for a clinical trial. As a matter of fact, the present study has to be considered as a pilot test. The research concerns the use of an antioxidant substance, the R(+)-thioctic acid, whose acute effects were previously tested on healthy subjects by some of us. In type II diabetic patients, the pilot test study consisted in a very long administration time and the wash out period. Despite the long administration time, none of the patients interrupted the trail so that the results could be obtained at each time from the whole subject sample. It is worth noting the great number of monitored parameters joining oxidative stress by using innovative instruments (EPR) applied by a mini-invasive technique and electroneurography investigations, together with the homogeneity of the assessed results, supported and strengthened by the significance of the correlations (see Figure 7), all suggesting the efficacy of the treatment, even if limited to a well-defined administration period (T0–T6). The behaviour leads us to hypothesize a typical hormetic trend. All together, these results can be regarded as a milestone in the field, particularly relevant in the suggestion that care must be adopted concerning the dose and the time of treatment by a substance suitable to prevent oxidative stress.

## 5. Conclusions

In type II DPN, alterations in the endogenous free radicals' scavenging defence mechanisms may lead to high ROS levels resulting in oxidative damage and nerve injury. In the present study, R(+)-thioctic acid 1.6 mg administration was shown to produce EPR detectable changes in ROS formation as measured in capillary blood and plasma and related oxidative damage parameters with consequent positive effects on nerve conduction velocity too. The findings of the present study strongly indicated that an increased antioxidant capacity plays an important role in the improvement of OxS, nerve conduction velocity, pain, and general wellness. Nevertheless, at the same time, the results of the study showed that the safety effects of an exogenous antioxidant compound were positive up to 60 administration days. After this period, a hormesis effect was observed. Therefore, the novelty of the conducted research would be a challenge for investigators to carefully address issues, including dose range factors, as well as appropriate administration time and targeting population to counteract possible “boomerang effects” of the treatment. The great number of parameters monitored in this study would firmly stress these conclusions.

## Figures and Tables

**Table 1:** Physical characteristics of subjects. Data are expressed as mean ± SD. BMI: body mass index; SaO_2_: arterial oxygen saturation; HR: heart rate.

Variable	Baseline data
Age (year)	66.4 ± 9.3
Weight (Kg)	70.4 ± 11.9
Height (m)	1.68 ± 0.03
BMI (Kg/m^2^)	24.7 ± 1.3
SaO_2_ (%)	96.55 ± 0.88
HR (BPM)	73.77 ± 11.93
Blood pressure (mmHg)	144 ± 11
Duration of diabetes (years)	17.4 ± 8.01

## Abbreviations

DPN: Diabetic peripheral neuropathy
OxS: Oxidative stress
EPR: Electron paramagnetic resonance
ROS: Reactive oxygen species
TAC: Antioxidant capacity
TBARS: Thiobarbituric acid reactive substances
PC: Protein carbonyls
BMI: Body mass index
SaO_2_: Arterial oxygen saturation
HR: Heart rate
RBCs: Red blood cells
HGB: Haemoglobin
HCT: Hematocrit
MCV: Mean corpuscular volume
MCH: Mean corpuscular haemoglobin
MCHC: Mean corpuscular haemoglobin concentration
RDW: Random distribution of red cell width
WBCs: White blood cells
%LYM: Lymphocyte percentage
%MON: Monocyte percentage
%GRA: Granulocyte percentage
#LYM: Lymphocyte number
#MON: Monocyte number
#GRA: Granulocyte number
PLT: Platelets
MPV: Mean platelet volume
LDH: Lactate dehydrogenase
S-lactate dehydrogenase: Serum lactate dehydrogenase
S-creatinine: Serum creatinine
CRP: C-reactive protein
HbA1c: Haemoglobin A1c
GI: Glycaemic index
DNS: Diabetic neuropathy symptom
PNP: Polyneuropathy
ENG: Electroneurography
MNCV: Motor nerve conduction velocity
SNCV: Sensory nerve conduction velocity
SPE: Superficial peroneal nerve
VAS: Visual analogue scale.

## References

[1] Wild S., Roglic G., Green A., Sicree R., King H. (2004). Global prevalence of diabetes: estimates for the year 2000 and projections for 2030. *Diabetes Care*.

[2] Sacks D. B., McDonald J. M. (1996). The pathogenesis of type II diabetes mellitus: *a polygenic disease*. *American Journal of Clinical Pathology*.

[3] Sas K. M., Kayampilly P., Byun J. (2016). Tissue-specific metabolic reprogramming drives nutrient flux in diabetic complications. *JCI Insight*.

[4] Hosseini A., Abdollahi M. (2013). Diabetic neuropathy and oxidative stress: therapeutic perspectives. *Oxidative Medicine and Cellular Longevity*.

[5] Tesfaye S., Selvarajah D. (2012). Advances in the epidemiology, pathogenesis and management of diabetic peripheral neuropathy. *Diabetes/Metabolism Research and Reviews*.

[6] Daousi C., Benbow S. J., Woodward A., MacFarlane I. A. (2006). The natural history of chronic painful peripheral neuropathy in a community diabetes population. *Diabetic Medicine*.

[7] Davies M., Brophy S., Williams R., Taylor A. (2006). The prevalence, severity, and impact of painful diabetic peripheral neuropathy in type 2 diabetes. *Diabetes Care*.

[8] Fiorentino T. V., Prioletta A., Zuo P., Folli F. (2013). Hyperglycemia-induced oxidative stress and its role in diabetes mellitus related cardiovascular diseases. *Current Pharmaceutical Design*.

[9] Hinder L. M., Vivekanandan-Giri A., McLean L. L., Pennathur S., Feldman E. L. (2013). Decreased glycolytic and tricarboxylic acid cycle intermediates coincide with peripheral nervous system oxidative stress in a murine model of type 2 diabetes. *The Journal of Endocrinology*.

[10] Valko M., Leibfritz D., Moncol J., Cronin M. T. D., Mazur M., Telser J. (2007). Free radicals and antioxidants in normal physiological functions and human disease. *The International Journal of Biochemistry & Cell Biology*.

[11] Bailey D. M., Lawrenson L., McEneny J. (2007). Electron paramagnetic spectroscopic evidence of exercise-induced free radical accumulation in human skeletal muscle. *Free Radical Research*.

[12] Mrakic-Sposta S., Gussoni M., Porcelli S. (2015). Training effects on ROS production determined by electron paramagnetic resonance in master swimmers. *Oxidative Medicine and Cellular Longevity*.

[13] Mrakic-Sposta S., Gussoni M., Montorsi M., Porcelli S., Vezzoli A. (2014). A quantitative method to monitor reactive oxygen species production by electron paramagnetic resonance in physiological and pathological conditions. *Oxidative Medicine and Cellular Longevity*.

[14] Sajfutdinov R. G., Larina L. I., Vakul’skaya T. I., Voronkov M. G. (2001). *Electron Paramagnetic Resonance in Biochemistry and Medicine*.

[15] Dikalov S. I., Dikalova A. E., Mason R. P. (2002). Noninvasive diagnostic tool for inflammation-induced oxidative stress using electron spin resonance spectroscopy and an extracellular cyclic hydroxylamine. *Archives of Biochemistry and Biophysics*.

[16] Tesfaye S., Boulton A. J. M., Dyck P. J. (2010). Diabetic neuropathies: update on definitions, diagnostic criteria, estimation of severity, and treatments. *Diabetes Care*.

[17] Javed S., Alam U., Malik R. A. (2015). Burning through the pain: treatments for diabetic neuropathy. *Diabetes, Obesity and Metabolism*.

[18] Herman IJzerman T., Schaper N. C., Melai T., Meijer K., Willems P. J. B., Savelberg H. H. C. M. (2012). Lower extremity muscle strength is reduced in people with type 2 diabetes, with and without polyneuropathy, and is associated with impaired mobility and reduced quality of life. *Diabetes Research and Clinical Practice*.

[19] Butugan M. K., Sartor C. D., Watari R. (2014). Multichannel EMG-based estimation of fiber conduction velocity during isometric contraction of patients with different stages of diabetic neuropathy. *Journal of Electromyography and Kinesiology*.

[20] Andreassen C. S., Jakobsen J., Andersen H. (2006). Muscle weakness: a progressive late complication in diabetic distal symmetric polyneuropathy. *Diabetes*.

[21] Nagamatsu M., Nickander K. K., Schmelzer J. D. (1995). Lipoic acid improves nerve blood flow, reduces oxidative stress, and improves distal nerve conduction in experimental diabetic neuropathy. *Diabetes Care*.

[22] Low P. A., Nickander K. K., Tritschler H. J. (1997). The roles of oxidative stress and antioxidant treatment in experimental diabetic neuropathy. *Diabetes*.

[23] Ziegler D., Nowak H., Kempler P., Vargha P., Low P. A. (2004). Treatment of symptomatic diabetic polyneuropathy with the antioxidant *α*-lipoic acid: a meta-analysis. *Diabetic Medicine*.

[24] Ziegler D., Hanefeld M., Ruhnau K. J. (1999). Treatment of symptomatic diabetic polyneuropathy with the antioxidant alpha-lipoic acid: a 7-month multicenter randomized controlled trial (ALADIN III study). ALADIN III study group. Alpha-lipoic acid in diabetic neuropathy. *Diabetes Care*.

[25] Ziegler D., Low P. A., Litchy W. J. (2011). Efficacy and safety of antioxidant treatment with *α*-lipoic acid over 4 years in diabetic polyneuropathy: the NATHAN 1 trial. *Diabetes Care*.

[26] Dyck P. J., Norell J. E., Tritschler H. (2007). Challenges in design of multicenter trials: end points assessed longitudinally for change and monotonicity. *Diabetes Care*.

[27] Javed S., Alam U., Malik R. A. (2015). Treating diabetic neuropathy: present strategies and emerging solutions. *The Review of Diabetic Studies*.

[28] Mijnhout G. S., Kollen B. J., Alkhalaf A., Kleefstra N., Bilo H. J. G. (2012). Alpha lipoic acid for symptomatic peripheral neuropathy in patients with diabetes: a meta-analysis of randomized controlled trials. *International Journal of Endocrinology*.

[29] Shakher J., Stevens M. J. (2011). Update on the management of diabetic polyneuropathies. *Diabetes, Metabolic Syndrome and Obesity: Targets and Therapy*.

[30] Devitt M. (2012). AAN, AANEM, and AAPMR publish guideline for treatment of painful diabetic neuropathy. *American Family Physician*.

[31] Derosa G., D’Angelo A., Romano D., Maffioli P. (2016). A clinical trial about a food supplement containing *α*-lipoic acid on oxidative stress markers in type 2 diabetic patients. *International Journal of Molecular Sciences*.

[32] American Diabetes Association (2016). Standards of medical care in diabetes - 2016. *Diabetes Care*.

[33] Meijer J. W. G., Smit A. J., Sonderen E. V., Groothoff J. W., Eisma W. H., Links T. P. (2002). Symptom scoring systems to diagnose distal polyneuropathy in diabetes: the diabetic neuropathy symptom score. *Diabetic Medicine*.

[34] Mrakic-Sposta S., Gussoni M., Montorsi M., Porcelli S., Vezzoli A. (2012). Assessment of a standardized ROS production profile in humans by electron paramagnetic resonance. *Oxidative Medicine and Cellular Longevity*.

[35] Mrakic-Sposta S., Gussoni M., Moretti S. (2015). Effects of mountain ultra-marathon running on ROS production and oxidative damage by micro-invasive analytic techniques. *PLoS One*.

[36] Collins S. L., Moore R. A., McQuay H. J. (1997). The visual analogue pain intensity scale: what is moderate pain in millimetres?. *Pain*.

[37] Golbidi S., Badran M., Laher I. (2011). Diabetes and alpha lipoic acid. *Frontiers in Pharmacology*.

[38] de Lemos E. T., Oliveira J., Pinheiro J. P., Reis F. (2012). Regular physical exercise as a strategy to improve antioxidant and anti-inflammatory status: benefits in type 2 diabetes mellitus. *Oxidative Medicine and Cellular Longevity*.

[39] Zimmer G., Fuchs J., Packer L., Zimmer G. (1997). Overview of the role of lipoate in the enzyme complexes of energy metabolism and reducing equivalents. *Lipoic Acid in Health and Diseases*.

[40] Packer L., Witt E. H., Tritschler H. J. (1995). Alpha-lipoic acid as a biological antioxidant. *Free Radical Biology and Medicine*.

[41] Ford I., Cotter M. A., Cameron N. E., Greaves M. (2001). The effects of treatment with [alpha ]-lipoic acid or evening primrose oil on vascular hemostatic and lipid risk factors, blood flow, and peripheral nerve conduction in the streptozotocin-diabetic rat. *Metabolism*.

[42] Wallace D. C. (2002). Animal models for mitochondrial disease. *Mitochondrial DNA*.

[43] Ceriello A., Bortolotti N., Motz E. (2001). Red wine protects diabetic patients from meal-induced oxidative stress and thrombosis activation: a pleasant approach to the prevention of cardiovascular disease in diabetes. *European Journal of Clinical Investigation*.

[44] Vega-López S., Devaraj S., Jialal I. (2004). Oxidative stress and antioxidant supplementation in the management of diabetic cardiovascular disease. *Journal of Investigative Medicine*.

[45] Altavilla D., Saitta A., Cucinotta D. (2001). Inhibition of lipid peroxidation restores impaired vascular endothelial growth factor expression and stimulates wound healing and angiogenesis in the genetically diabetic mouse. *Diabetes*.

[46] Greene D. A., Stevens M. J., Obrosova I., Feldman E. L. (1999). Glucose-induced oxidative stress and programmed cell death in diabetic neuropathy. *European Journal of Pharmacology*.

[47] Villegas-Rivera G., Román-Pintos L. M., Cardona-Muñoz E. G. (2015). Effects of ezetimibe/simvastatin and rosuvastatin on oxidative stress in diabetic neuropathy: a randomized, double-blind, placebo-controlled clinical trial. *Oxidative Medicine and Cellular Longevity*.

[48] Calabrese V., Cornelius C., Trovato-Salinaro A. (2010). The hormetic role of dietary antioxidants in free radical-related diseases. *Current Pharmaceutical Design*.

[49] Calabrese E. J., Baldwin L. A. (2003). Hormesis: the dose-response revolution. *Annual Review of Pharmacology and Toxicology*.

[50] Calabrese E. J. (2008). Hormesis and medicine. *British Journal of Clinical Pharmacology*.

[51] Mandel S., Amit T., Reznichenko L., Weinreb O., Youdim M. B. H. (2006). Green tea catechins as brain-permeable, natural iron chelators-antioxidants for the treatment of neurodegenerative disorders. *Molecular Nutrition & Food Research*.

[52] Cook R., Calabrese E. J. (2006). The importance of hormesis to public health. *Environmental Health Perspectives*.

[53] Rattan S. I. S. (2008). Hormesis in aging. *Ageing Research Reviews*.

[54] Halliwell B. (2008). Are polyphenols antioxidants or pro-oxidants? What do we learn from cell culture and *in vivo* studies?. *Archives of Biochemistry and Biophysics*.

[55] Karasu Ç., Dewhurst M., Stevens E. J., Tomlinson D. R. (1995). Effects of anti-oxidant treatment on sciatic nerve dysfunction in streptozotocin-diabetic rats; comparison with essential fatty acids. *Diabetologia*.

[56] Nickander K. K., Schmelzer J. D., Rohwer D. A., Low P. A. (1994). Effect of *α*-tocopherol deficiency on indices of oxidative stress in normal and diabetic peripheral nerve. *Journal of the Neurological Sciences*.

[57] Vallianou N., Evangelopoulos A., Koutalas P. (2009). Alpha-lipoic acid and diabetic neuropathy. *The Review of Diabetic Studies*.

[58] Seyit D. A., Degirmenci E., Oguzhanoglu A. (2016). Evaluation of electrophysiological effects of melatonin and alpha lipoic acid in rats with streptozotocine induced diabetic neuropathy. *Experimental and Clinical Endocrinology & Diabetes*.

[59] Nickander K. K., Mcphee B. R., Low P. A., Tritschler H. (1996). Alpha-lipoic acid: antioxidant potency against lipid peroxidation of neural tissues in vitro and implications for diabetic neuropathy. *Free Radical Biology & Medicine*.

[60] Stevens M. J., Obrosova I., Cao X., Van Huysen C., Greene D. A. (2000). Effects of DL-alpha-lipoic acid on peripheral nerve conduction, blood flow, energy metabolism, and oxidative stress in experimental diabetic neuropathy. *Diabetes*.

[61] Al-Nimer M. S., Al-Ani F. S., Ali F. S. (2012). Role of nitrosative and oxidative stress in neuropathy in patients with type 2 diabetes mellitus. *Journal of Neurosciences in Rural Practice*.

[62] Rota E., Morelli N. (2016). Entrapment neuropathies in diabetes mellitus. *World Journal of Diabetes*.

[63] Negrişanu G., Roşu M., Bolte B., Lefter D., Dabelea D. (1999). Effects of 3-month treatment with the antioxidant alpha-lipoic acid in diabetic peripheral neuropathy. *Romanian Journal of Internal Medicine*.

[64] Melzack R., Katz J. (2013). Pain. *Wiley Interdisciplinary Reviews: Cognitive Science*.

[65] Ziegler D., Ametov A., Barinov A. (2006). Oral treatment with *α*-lipoic acid improves symptomatic diabetic polyneuropathy. *Diabetes Care*.

